# Machine learning methods for developments of binding kinetic models in predicting protein‐ligand dissociation rate constants

**DOI:** 10.1002/smo.20230012

**Published:** 2023-11-10

**Authors:** Yujing Zhao, Qilei Liu, Jian Du, Qingwei Meng, Lei Zhang

**Affiliations:** ^1^ State Key Laboratory of Fine Chemical Frontiers Science Center for Smart Materials Oriented Chemical Engineering Institute of Chemical Process Systems Engineering School of Chemical Engineering Dalian University of Technology Dalian China; ^2^ Ningbo Institute of Dalian University of Technology Ningbo China

**Keywords:** Bayesian neural network, binding kinetics, dissociation rate constant, machine learning, protein‐ligand interaction energies

## Abstract

Binding kinetic properties of protein–ligand complexes are crucial factors affecting the drug potency. Nevertheless, the current *in silico* techniques are insufficient in providing accurate and robust predictions for binding kinetic properties. To this end, this work develops a variety of binding kinetic models for predicting a critical binding kinetic property, dissociation rate constant, using eight machine learning (ML) methods (Bayesian Neural Network (BNN), partial least squares regression, Bayesian ridge, Gaussian process regression, principal component regression, random forest, support vector machine, extreme gradient boosting) and the descriptors of the van der Waals/electrostatic interaction energies. These eight models are applied to two case studies involving the HSP90 and RIP1 kinase inhibitors. Both regression results of two case studies indicate that the BNN model has the state‐of‐the‐art prediction accuracy (HSP90: $R_{\text{test}}^{2}=0.947$, *MAE*_test_ = 0.184, *r*_test_ = 0.976, *RMSE*_test_ = 0.220; RIP1 kinase: $R_{\text{test}}^{2}=0.745$, *MAE*_test_ = 0.188, *r*_test_ = 0.961, *RMSE*_test_ = 0.290) in comparison with other seven ML models.

## Graphical abstract

This paper develops a variety of binding kinetic models for predicting dissociation rate constants using eight machine learning methods, which are tested by two case studies involving the HSP90 and RIP1 kinase inhibitors. Both regression results of the two case studies indicate that the Bayesian neural network model has the state‐of‐the‐art prediction accuracy in comparison with the other seven machine learning algorithms.

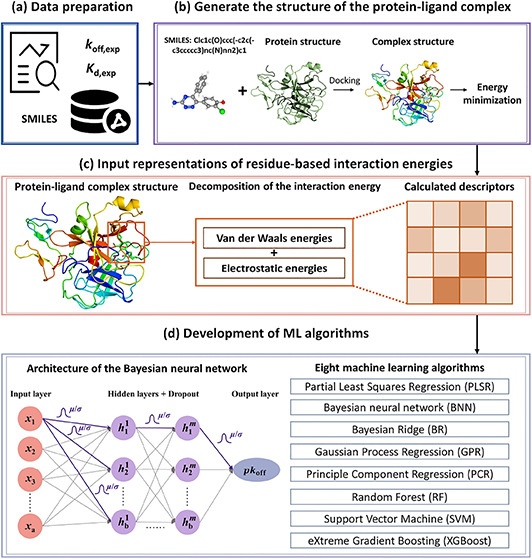

## 1 INTRODUCTION

Small molecule drugs play a significant role in preventing disease and protecting public health.^[1]^ However, there are a number of challenges that hamper the development of innovative drugs, including the high cost of bringing a drug to the market, the low success rate in clinical trials, and the lengthy development cycle. The average cost of developing a novel drug is estimated to be over two billion dollars and the development process takes approximately 10–15 years.^[2]^ A recent paradigm shift in drug discovery has emphasized the importance of kinetic properties (e.g., the dissociation rate constant (*k*_off_), the protein–ligand residence time (*τ*, *τ* = 1/*k*_off_), etc.) as a key goal in addition to the strong binding affinity. The importance of kinetic properties for drug potency has recently been increasingly recognized, and the kinetic properties are gradually being used in the optimization and design of candidate drugs.[^3^, ^4^, ^5^, ^6^, ^7^]

Finding a fast and reliable method to obtain the kinetic properties of protein–ligand interactions has been an important task in drug discovery. In kinetic measurements, the application of conventional experimental methods is challenging due to the fact that the binding/unbinding rate constants are dependent on the Gibbs free energies of intermediate transition states,^[8]^ and binding/unbinding processes generally take several minutes or even days, which makes it difficult to observe the experimental response using conventional analysis methods such as the X‐ray crystallography or the nuclear magnetic resonance. Currently, the kinetic properties are mainly determined by biophysical techniques such as isothermal titration calorimetry,^[9]^ capillary electrophoresis,^[10]^ affinity chromatography,^[11]^ and Surface Plasmon Resonance (SPR) methods.[^12^, ^13^, ^14^] For instance, the SPR spectroscopy is commonly used on flexible platforms. However, it is generally not suitable for detecting fast association or dissociation kinetics.^[15]^ As a complementary technique to SPR spectroscopy, the affinity chromatography is able to increase reproducibility and reduce cost per analysis. Capillary electrophoresis is advantageous for its efficiency, speed, and low sample requirement.^[15]^ In spite of the above methods, there are still technical difficulties associated with each method (e.g., slowness, errors in measurements, high costs),[^16^, ^17^, ^18^] especially in high‐throughput measurements. For this reason, computational methods are paid more and more attention to provide insights into the protein–ligand binding/unbinding processes.

The Molecular Dynamics (MD) simulations are widely used to analyze the dissociation pathway of the ligand from the protein in atom‐level detail.[^19^, ^20^, ^21^] Considering that the *τ* of pharmaceutically interesting compounds (minutes to hours) in reality are far beyond the time simulated using the conventional MD techniques (μs), the simulation of unbinding rates is therefore tremendously challenging if only conventional MD is used.^[19]^ Hitherto, MD simulations are used only for molecules with fast dissociation rates (typically with *k*_off_ > 10^3^ s^−1^) to obtain their unbinding rates.[^20^, ^22^, ^23^, ^24^] This limitation has motivated the development of novel MD sampling algorithms by adding biasing potentials along selected collective variables (e.g., using metadynamics[^25^, ^26^] or selecting reaction‐relevant regions of protein–ligand configurational space (e.g., by the adaptive multilevel splitting method^[27]^ or weighted ensemble sampling.[^8^, ^28^, ^29^, ^30^] For example, Kokh et al.^[31]^ proposed a protocol to predict the *τ* values of 70 inhibitors of human Heat Shock Protein 90*α* (HSP90*α*) through the *τ*‐Random Acceleration Molecular Dynamics (*τ‐*RAMD). There is an acceptable correlation (determination coefficient (*R* ^2^) = 0.66) between the predicted and measured *τ* in 59 samples after removing 11 samples. Further exclusion of four outlier compounds resulted in the *R* ^2^ of 0.86 and the Mean Absolute Percentage Error (*MAPE*) of 0.36. Further testing of the prediction method was conducted using 94 HSP90 inhibitors. The results showed that the predicted *R* ^2^ of the 80 inhibitors was 0.75 with the *MAPE* of 0.39.^[32]^ Even though the *τ‐*RAMD simulations are able to provide a feasible method for predicting protein–ligand binding kinetics, their practical effectiveness is limited by the amount of MD computational resources and the inadequacy of MD force fields.

In addition to the above mechanism models, surrogate modeling methods have recently received more and more attention owing to their efficiency. The Molecular Mechanics (MM) ‐derived descriptors such as the protein–ligand interaction energy fingerprints are generally used to study the protein–ligand binding kinetics. For example, Chiu and Xie^[33]^ employed the Random Forest (RF) predictive clustering technique to develop a Machine Learning (ML) model based on the energetic and conformational dynamic descriptors generated from the MM method. The authors classified 39 Human Immunodeficiency Virus type 1 (HIV‐1) protease inhibitors into four classes according to their kinetic rate constants and achieved an accuracy of 74.35% in their model. Similarly, Zhang et al.^[34]^ decomposed the protein–ligand interaction fingerprints alone of the ligand‐unbinding pathway and constructed Partial Least Squares Regression (PLSR) models to predict the *pk*_off_ (‐log*k*_off_) values of 20 p38 mitogen‐activated protein kinase Type II inhibitors. The results showed that the *R* ^2^ on the training set (*R* ^2^_train_), the cross‐validated determination coefficient on the validation set (*Q* ^2^_cv_), and the *R* ^2^ on the test set (*R* ^2^_test_) of the optimal model with three descriptors are 0.720, 0.660, and 0.563, respectively. Additionally, three‐dimensional quantitative structure‐activity relationship studies have been carried out to predict the kinetic properties of HIV‐1 protease inhibitors. Using the comparative molecular field analysis method, Schaal^[35]^ predicted *pk*_off_ for 34 HIV‐1 protease inhibitors. After a laborious process of molecular alignment and parameter optimization, the optimal *pk*_off_ model yielded a *Q* ^2^_cv_ of 0.72 and an *R* ^2^_test_ of 0.60. In addition, 37 HIV‐1 protease inhibitors were analyzed using the PLSR method using the VolSurf descriptors.^[15]^ The results showed that the *R* ^2^_train_, *Q* ^2^_cv_, and *R* ^2^_test_ of the optimal model are 0.695, 0.695, and 0.772, respectively. More similar studies can be found in literature.[^36^, ^37^, ^38^, ^39^, ^40^] Some surrogate models that involve multiple proteins have also been developed. For example, Fedorov et al.^[41]^ constructed a database containing 501 protein–ligand complexes with *k*_off_ collected from the public literature. Then, they utilized their dataset to develop an RF model for predicting *pk*_off_ with *R* ^2^ = 0.60. Liu et al.^[42]^ established a database consisting of 680 experimental *k*_off_ and the corresponding protein–ligand complex structures. Then, the authors derived a general RF model based on this database to predict *pk*_off_ for multiple proteins. The final optimal model was selected with the Pearson correlation coefficient (*r*) of 0.968 and the Root Mean Square Error (*RMSE*) of 0.474 on the training set, as well as the *r* of 0.501 and the RMSE of 0.891 on the HSP90 test set.

Even with these exploratory studies, the prediction accuracy of current surrogate models remains bottlenecks for predicting binding kinetic properties. To address this issue, eight ML models, including Bayesian Neural Network (BNN), PLSR, Bayesian Ridge (BR), Gaussian Process Regression (GPR), Principal Component Regression (PCR), RF, Support Vector Machine (SVM), and eXtreme Gradient Boosting (XGBoost), are developed for estimations of *pk*_off_ using two types of MM‐derived descriptors determined from the energy‐minimized structures of protein–ligand complexes. The developed BNN‐based binding kinetic model achieved the state‐of‐the‐art prediction accuracy for estimating *pk*_off_ in comparison with other commonly used ML models. This paper is organized as follows. In the second section, the binding kinetic modeling methodology is discussed in detail, including the data preparation, the acquisition of energy‐minimized structures of protein–ligand complexes, the input representation of residue‐based interaction energies (descriptor), and the developments of the ML models. In the third section, two case studies involving the HSP90 and RIP1 kinase inhibitors are used to test the binding kinetic modeling methodology. The prediction performances of various ML models on *pk*_off_ are also compared and discussed. It is expected that the model developed in this work can be used to screen potential ligands with the desired *k*_off_ for the HSP90 and RIP1 kinase proteins. Also, the methodology proposed in this paper is able to provide a useful reference for predicting *k*_off_ of the molecules that bind to other proteins, as well as other binding kinetic properties.

## 2 BINDING KINETIC MODELING METHODOLOGY

**FIGURE 1:**
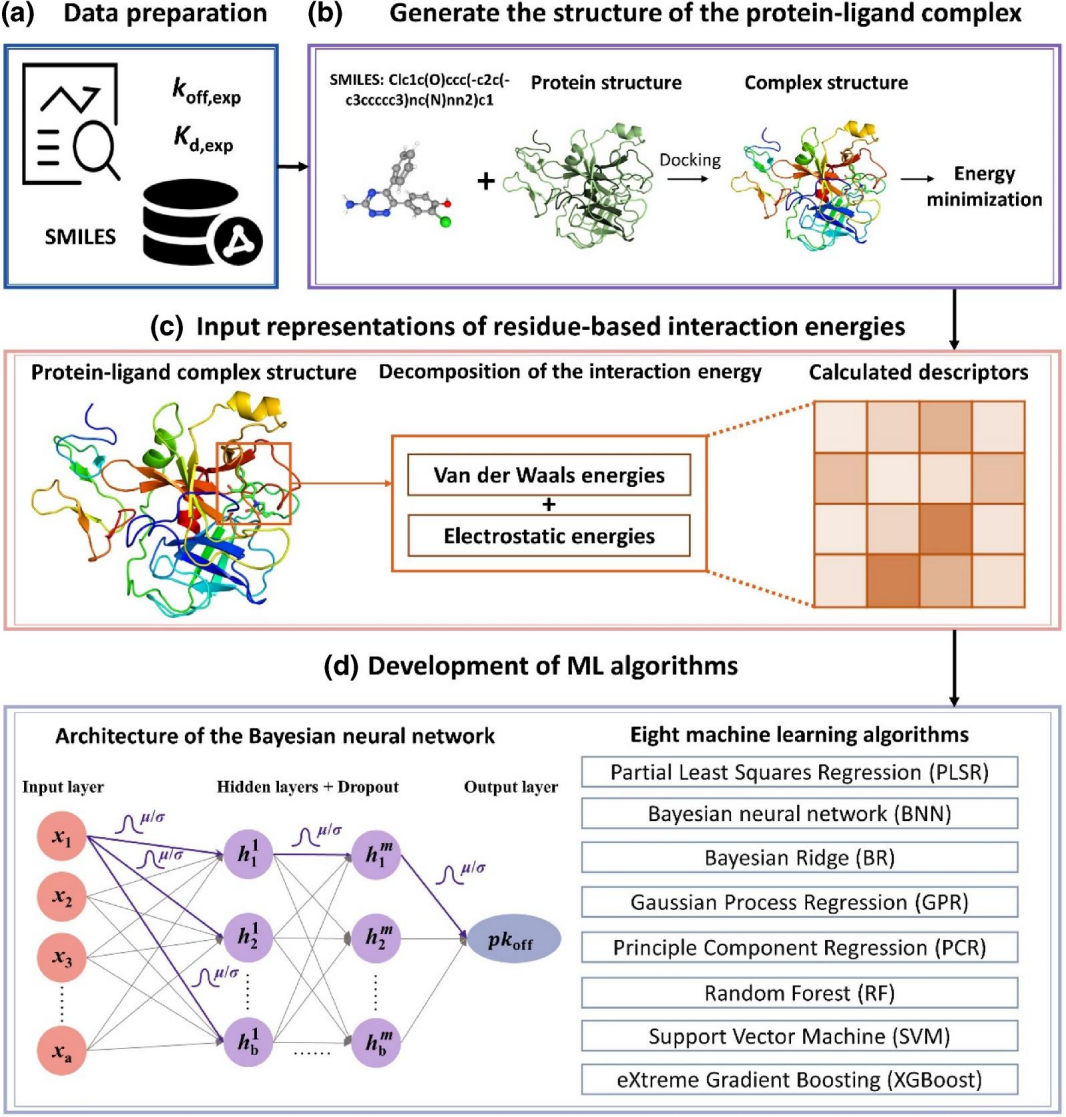
The workflow of the binding kinetic modeling methodology.

In this section, a binding kinetic modeling methodology is proposed to develop ML‐based binding kinetic models for estimations of *pk*_off_. Figure 1 depicts the workflow of the computational procedure in this study, which mainly consists of four stages.

In the first stage (Figure 1a), a database is established containing the binding kinetics and affinity data of a number of ligands for a certain protein. In the second stage (Figure 1b), the three‐dimensional structures of protein‐ligand complexes are collected for each kinetic data point from protein databases or via the docking method, and then the energy minimizations are carried out for the obtained three‐dimensional structures of protein‐ligand complexes. Then, in the third stage (Figure 1c), the input representations (descriptors) of the residue‐based interaction energies (both van der Waals and electrostatic energies) are calculated by the gCOMBINE software^[43]^ based on the energy‐minimized structures of complexes. In the fourth stage (Figure 1d), eight ML models are developed via corelating the experimental *pk*_off_ with a certain number of principal components, which are derived from the descriptors of the interaction energies through data pretreatments including the Principal Component Analysis (PCA) method. More details for these four stages are described in the following subsections.

### 2.1 Data preparation

Current public collections of protein–ligand kinetics data are comparably rarer than the affinity data. Some well‐known public databases (e.g., Binding MOAD,^[44]^ etc.) that collect the protein–ligand binding affinity data have not included kinetics‐related data. This is why the published kinetics studies have focused on the datasets with very few proteins. Currently, there are some databases that contain kinetics data, including KDBI,[^45^, ^46^] KOFFI,^[47]^ BindingDB,^[48]^ PDBbind‐koff‐2020,^[42]^ KINetic Dataset (KIND),^[49]^ and so on.

To the best of our knowledge, KIND^[49]^ is the largest publicly available kinetics dataset so far, comprising a total of 3812 small molecule structures with their association rate constants (*k*_on_), *k*_off_, and *K*_d_. It has been compiled from 21 publications and the K4DD database. For these publications, only the papers containing numerical values for all three properties investigated (*k*_on_, *k*_off_, *K*_d_) are selected. The K4DD consortium merges the efforts of 22 partners from European academia and the pharmaceutical industry in order to explore the role of kinetics in drug discovery. The kinetics data points collected are mainly derived from SPR experiments, radioligand binding assays, and so on. This results in 3812 small molecules annotated to 78 different proteins from five protein classes, comprising 3238 *k*_on_, *k*_off_, *K*_d_ for kinases, 242 for G protein‐coupled receptors, 160 for HSPs, 127 for enzymes, and 45 for ion channels.

In this work, two critical proteins, HSP90 and RIP1 kinase, are considered to test our proposed methodology. The HSP90 protein is a highly abundant and ubiquitous molecular chaperone that plays an essential role in many cellular processes. It has become a major therapeutic protein for cancer, and there has also been increasing interest in it as a therapeutic protein in neurodegenerative disorders and in the development of anti‐virals and anti‐protozoan infections.^[50]^ The RIP1 kinase protein has emerged as a key upstream regulator that controls inflammatory signaling as well as the activation of multiple cell death pathways, including apoptosis and necroptosis.^[51]^ Targeting RIP1 kinase might provide novel therapeutics for the treatment of both acute and chronic human diseases.^[51]^

### 2.2 Obtain energy‐minimized three‐dimensional structures of protein–ligand complexes

After choosing the suitable dataset, the next step is to prepare a set of energy‐minimized three‐dimensional structures of protein–ligand complexes in the dataset. Protein‐ligand complexes whose crystal structures have not yet been determined are generally docked to obtain their binding poses, taking into account the preparation time and cost. Thus, three tasks of molecular docking, electric charge calculation, and energy minimization need to be carried out in turn.

#### 2.2.1 Molecular docking

The three‐dimensional conformation of the ligand is docked to the protein using the AutoDock Vina software.^[52]^ For each complex, 20 ligand poses are set to be generated by the docking method, and the top‐ranked pose is selected for further energy minimization. Parameters for docking are optimized by redocking, resulting in the choice of an exhaustiveness level of 24 and a cubic grid with a spacing of 0.375 Å and 30 Å along each edge centered in the known binding site. Bond rotations are allowed in ligands, while the protein structures are kept rigid.

#### 2.2.2 Electric charge calculation

After the docking method, the hydrogen atoms are added to the complexes. The Restrained ElectroStatic Potential 2 (RESP2)^[53]^ partial atomic charges for each ligand are calculated using the electrostatic potentials from quantum chemistry‐based calculations using the Gaussian^[54]^ and Multiwfn software.^[55]^

#### 2.2.3 Energy minimization

Energy minimization is performed for protein–ligand complexes through two steps. In the first step, a harmonic restraint with a force constant of 100 kcal/(mol·Å^2^) is applied to the positions of the heavy atoms. In the second step, no positional restraint is used. For each step of energy minimization, 1000 steps of the steepest descent algorithm followed by 4000 steps of the conjugate gradient algorithm are applied. Energy minimization is performed using an implicit solvent environment. Constructed force field parameters and topology files are used for system energy minimizations through the AMBER software.^[56]^ Parameters to describe the protein and the ligand are obtained from the AMBER ff14SB force field^[57]^ and GAFF,^[58]^ respectively.

### 2.3 Input representation calculation

Based on the energy‐minimized three‐dimensional structures of protein–ligand complexes, the input representations (the residue‐based van der Waals and electrostatic interaction energies) of the protein‐ligand complexes in datasets for ML modeling are calculated. The interaction energies of residue‐ligand pairs are obtained by decomposing the total energy of a protein–ligand complex into the van der Waals and electrostatic interaction energies between the ligand and each protein residue, as shown in Equation (1). These energies are computed from the energy‐minimized structures of the complexes through the gCOMBINE software^[43]^ and the AMBER ff14SB force field.^[57]^ Electrostatic interactions are computed using a uniform dielectric constant of 4.

(1) $$E=\sum_{k=1}^{n_{p}}E_{k}^{\text{vdW}}+\sum_{k=1}^{n_{p}}E_{k}^{\text{elec}}$$

where *E* ^vdW^ and *E* ^elec^ are van der Waals and electrostatic interaction energies, respectively, *n*_p_ is the total number of protein residues, and *k* is the index of residues. Finally, the input representations of the protein–ligand complexes are represented by an **X** matrix, as shown in Equation (2).

(2) $$X=\left[\begin{array}{ccccccccc} E_{1,1}^{\text{vdW}} & E_{2,1}^{\text{vdW}} & \text{⋯} & E_{n_{p},1}^{\text{vdW}} & E_{1,1}^{\text{elec}} & E_{2,1}^{\text{elec}} & \text{⋯} & \text{⋯} & E_{n_{p},1}^{\text{elec}}\\ E_{1,2}^{\text{vdW}} & E_{2,2}^{\text{vdW}} & \text{⋯} & E_{n_{p},2}^{\text{vdW}} & E_{1,2}^{\text{elec}} & E_{2,2}^{\text{elec}} & \text{⋯} & \text{⋯} & E_{n_{p},2}^{\text{elec}}\\ & & \text{⋯} & & & & \text{⋯} & \text{⋯} & \\ E_{1,N}^{\text{vdW}} & E_{2,N}^{\text{vdW}} & \text{⋯} & E_{n_{p},N}^{\text{vdW}} & E_{1,N}^{\text{elec}} & E_{2,N}^{\text{elec}} & \text{⋯} & \text{⋯} & E_{n_{p},N}^{\text{elec}} \end{array}\right]$$

where *N* is the number of ligands. Other properties, superscripts, and subscripts have been explained above.

### 2.4 Developments of ML models

With the obtained experimental *pk*_off_ values and the input representations, the next step is to develop ML models for the predictions of *pk*_off_ through Equation (3). But before developing ML models, the data pretreatment consisting of four steps is performed for the **X** matrix.

(3) $$Y=\left[\begin{array}{c} \begin{array}{c} pk_{\text{off},1}\\ pk_{\text{off},2} \end{array}\\ \begin{array}{c} \text{⋯}\\ pk_{\text{off},N} \end{array} \end{array}\right]=f_{\text{ML}}\left(f_{\text{pretreat}}\left(X\right)\right)$$

#### 2.4.1 Data pretreatment

In the first step, positive van der Waals and electrostatic interaction energies, which may result from inconsistencies in the force field or errors in the model, are truncated to zero in this work. The second step (feature selection procedure) involves reducing the number of variables while maintaining the interaction energies of key protein regions in the **X** matrix by removing interaction energy values whose standard deviation is below a threshold. In the third step, the standardization scaling method is optionally employed to process the **X** matrix. In the fourth step, the PCA method (feature extraction procedure) is used to process the **X** matrix to further reduce the number of variables, keep the main information within the **X** matrix, and avoid overfitting issues of the subsequent ML modeling to some extent. Note that whether the standardization scaling method is used, as well as the number of principal components and the threshold of standard deviation, is determined according to different dataset studies.

#### 2.4.2 Machine learning models

In this work, eight ML methods, including PLSR, BNN, BR, GPR, PCR, RF, SVM and XGBoost, are employed to develop regression models for predictions of *pk*_off_, where PLSR is the most commonly used modeling method in literature. The BNN model is developed by our own codes based on the Python language,^[59]^ while the other seven ML models are available in the scikit‐learn library^[60]^ based on the Python language.^[59]^ Below, we briefly describe each model that has been used in our research.

PLSR is capable of decreasing the descriptor size to a smaller set consisting of uncorrelated components and performing the least squares regression on these components. It is applicable to the cases when the number of descriptors is larger than that of labels, or when the descriptors are highly collinear.

BNN combines the standard neural network with the Bayesian inference, where the weights and outputs are treated as the variables to find the marginal distributions that best fit the data. It is capable of quantifying the level of uncertainty in its predictions. As opposed to traditional neural networks, BNN uses marginalization to train the weights of the model as a probability distribution rather than seeking a single optimal point for representing a weight. The generic architecture of the BNN model is shown in Figure 1d.

BR combines the Bayesian and ridge regression methods. The Bayesian method provides a probabilistic model for the regression problem. The ridge regression method is able to develop a linear regression model whose coefficients are estimated by a ridge estimator.

GPR is a kernel‐based nonparametric, Bayesian approach to regression. It is able to work on small datasets, and provide uncertainty predictions. The kernel functions play an important role in the prediction accuracy of GPR and the linearity of GPR depends on the kernel functions.

PCR first uses the PCA method to transform the original descriptors into principal components, which are then used to develop the linear regression model. PCR is often used in the case when the descriptors are highly correlated.

RF is an ensemble technique that is able to perform regression tasks through the use of multiple decision trees in predicting the final output rather than relying on individual decision trees.

SVM is an ML model that is able to perform regression tasks. Its optimization goal is to identify a regression plane and make all data closest to the plane. A variety of complex problems are able to be addressed by applying different kernel functions in the SVM. The linearity of the SVM depends on the kernel functions.

XGBoost is an ML model under the Gradient Boosting framework. It provides a parallel tree boosting and is able to deal with many scientific modeling issues in an accurate and fast manner. Compared with other similar methods, the second‐order Taylor expansion is used in the loss function of XGBoost.

### 2.5 Performance evaluations for ML models

To ensure the consistency of model comparisons, the same initial descriptor set and dataset split are employed to implement the above eight ML models for each protein dataset. The samples in the dataset are ranked from low to high *pk*_off_ values and every fifth sample in the list is selected for the test set, that is, the protein–ligand complexes are divided into the training and test sets with the radio of 4:1. The training set is further trained and validated through the leave‐one‐out cross‐validation method, where the whole training set is split into equal‐sized parts and the number of parts equals to the number of samples in the training set. The prediction result of the only remaining part is calculated using the model that is trained with the remaining parts. The process is repeated a number of times equal to the number of training set samples so that every part is used as the validation set. More details of the cross‐validation method can be found in Rubén et al.^[43]^ All the above eight ML models use the knowledge‐based trial and leave‐one‐out cross‐validation methods to optimize their respective hyper‐parameters. The generalization capability of the trained promising model is evaluated by the test set.

A total number of 12 statistical indicators are used to evaluate the quality of the regression models, including the *R* ^2^ on the training set ($R_{\text{train}}^{2}$), the leave‐one‐out cross‐validated determination coefficient on the validation set ($Q_{\text{cv}}^{2}$), the *R* ^2^ on the test set ($R_{\text{test}}^{2}$), as well as the *r*, Mean Absolute Error (*MAE*), and *RMSE* on the training/validation/test sets (*r*_train_/*r*_cv_/*r*_test_, *MAE*_train_/*MAE*_cv_/*MAE*_test_, and *RMSE*_train_/*RMSE*_cv_/*RMSE*_test_, respectively). These statistical definitions are given in Equations ((4), (5), (6), (7)).

(4) $${R_{\text{train}/\text{test}}^{2}=Q}_{\text{cv}}^{2}=1-\frac{\sum_{i=1}^{M}{\left(y_{i,\exp}-y_{i,\text{pred}}\right)}^{2}}{\sum_{i=1}^{M}{\left(y_{i,\exp}-{\bar{y}}_{\exp}\right)}^{2}}$$

(5) $$r_{\text{train}/\text{cv}/\text{test}}=\frac{\sum_{i=1}^{M}\left(y_{i,\exp}-{\bar{y}}_{\exp}\right)\left(y_{i,\text{pred}}-{\bar{y}}_{\text{pred}}\right)}{\sum_{i=1}^{M}\left(y_{i,\exp}-{\bar{y}}_{\exp}\right)\sum_{i=1}^{M}\left(y_{i,\text{pred}}-{\bar{y}}_{\text{pred}}\right)}$$

(6) $${MAE}_{\text{train}/\text{cv}/\text{test}}=\frac{1}{M}\sum_{i=1}^{M}\left|y_{i,\exp}-y_{i,\text{pred}}\right|$$

(7) $${RMSE}_{\text{train}/\text{cv}/\text{test}}=\sqrt{\frac{\sum_{i=1}^{M}{\left(y_{i,\exp}-y_{i,\text{pred}}\right)}^{2}}{M}}$$

where *y*_*i*,exp_ is the experimental data of sample *i*, *y*_*i*,pred_ is the predicted data of sample *i*, ${\bar{y}}_{\exp}$ is the average experimental data of *M* samples (${\bar{y}}_{\exp}=\left(\sum_{i}^{M}y_{i,\exp}\right)/M$), ${\bar{y}}_{\text{pred}}$ is the average predicted data of *M* samples (${\bar{y}}_{\text{pred}}=\left(\sum_{i}^{M}y_{i,\text{pred}}\right)/M$). Generally, a statistical model with $Q_{\text{cv}}^{2}\geq 0.5$ is required for extrapolations.^[61]^

## 3 RESULTS AND DISCUSSIONS

This work develops a binding kinetic modeling methodology to predict *pk*_off_ using eight different ML methods. In this section, two critical proteins, HSP90 and RIP1 kinase, are taken as examples to test the proposed methodology. The HSP90 dataset is collected from Ganotra et al.,^[39]^ consisting of 66 protein–ligand complexes, together with their binding kinetics data, binding affinity data, and three‐dimensional structures of the energy‐minimized complexes. The RIP1 kinase dataset is collected from the KIND database,^[49]^ consisting of 35 protein–ligand complexes, together with their binding kinetics data and binding affinity data. Note that few crystallographic data are available for the RIP1 kinase–ligand complexes. Therefore, the docking and energy minimization methods are employed to obtain the three‐dimensional structures of RIP1 kinase–ligand complexes. In the following, the modeling results and discussions will be presented in detail.

### 3.1 Dataset quality analysis

**FIGURE 2:**
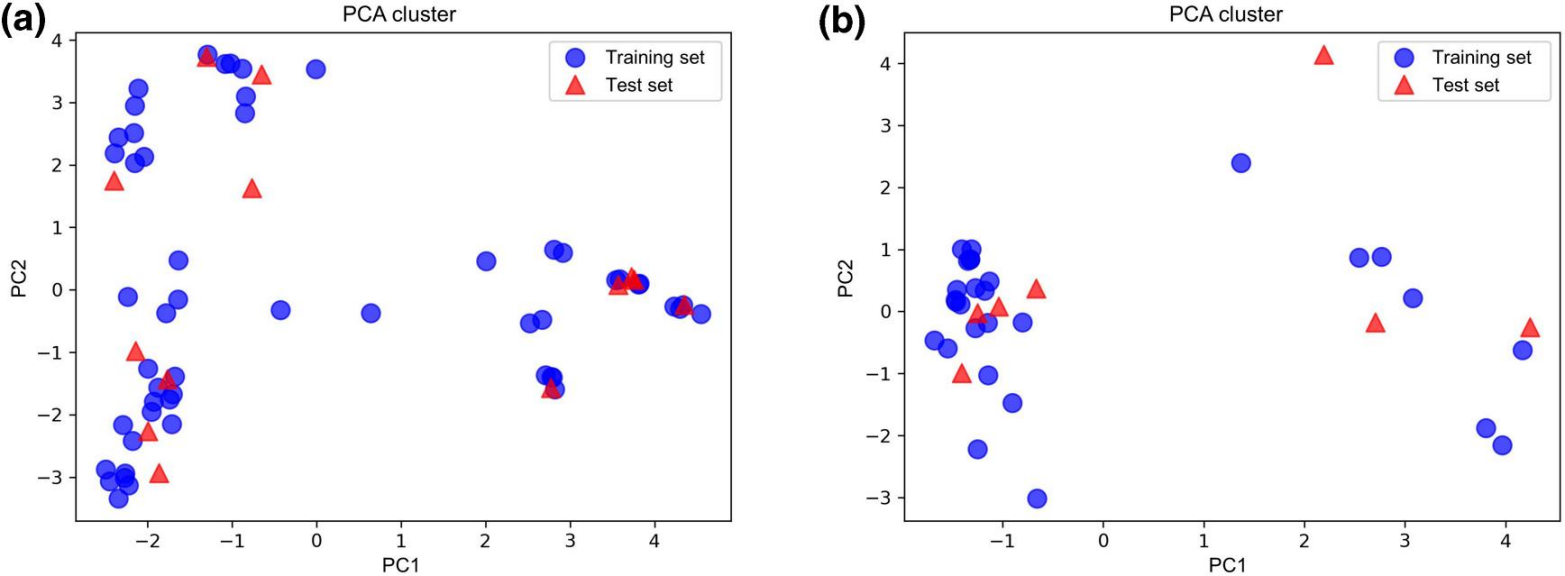
Molecular sparsity for the HSP90 and RIP1 kinase datasets.

**FIGURE 3:**
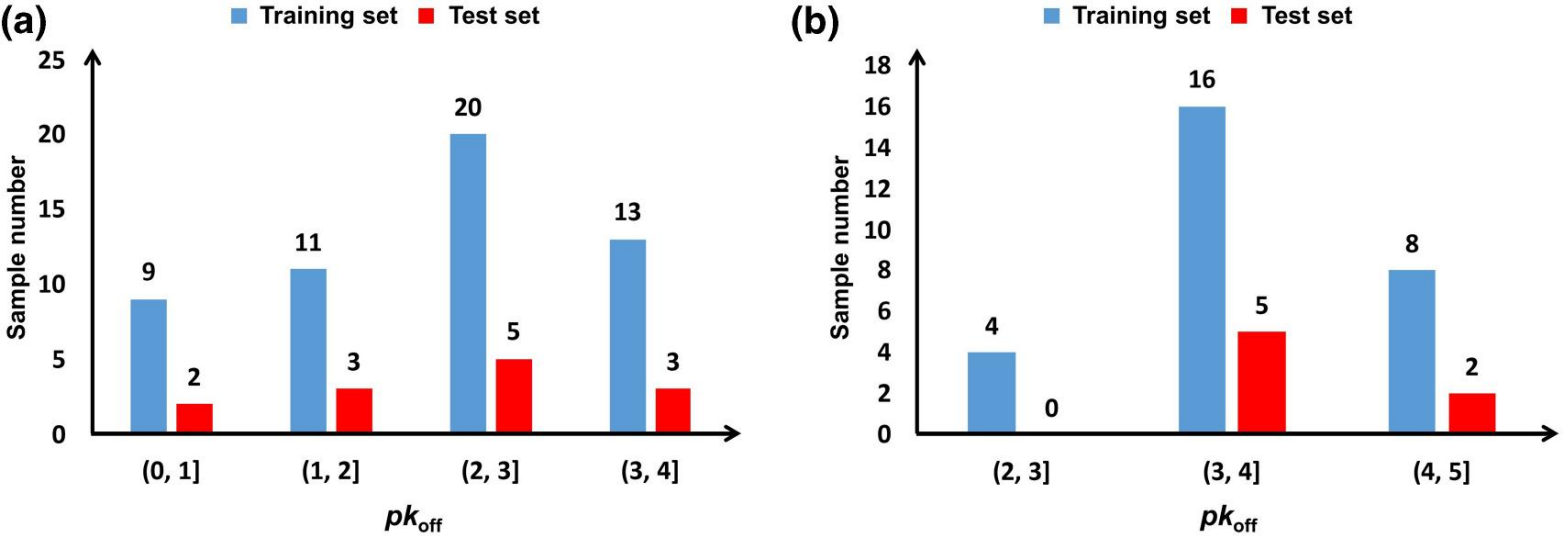
Label distributions for the HSP90 and RIP1 kinase datasets.

First, the quality of the HSP90 and RIP1 kinase datasets is evaluated, as shown in Figures 2 and 3. The chemical space of the ligands in the HSP90 dataset are not sparsely distributed (Figure 2a), which is desirable for ML modeling. The *x*‐axis and *y*‐axis of the chemical space (i.e., two principal components of PC1 and PC2) are obtained by using the extended‐connectivity fingerprints and the PCA method. For the RIP1 kinase dataset, the chemical space of the ligands in the RIP1 kinase dataset (Figure 2b) are not distributed as well as that of the ligands in the HSP90 dataset (Figure 2a). The use of sparsely distributed molecules (i.e., outliers) in chemical space for model training might diminish the model performance.

Besides, the values of *pk*_off_ in the HSP90 dataset are well‐distributed from 0 to 4 (Figure 3a). However, the distribution of the *pk*_off_ values in the RIP1 kinase dataset (Figure 3b) are comparatively worse than that in the HSP90 dataset (Figure 3a). The quality of the ML models strongly depends on the range and the homogeneity of the distribution of *pk*_off_ for the compounds studied. The *k*_off_ distribution would be better to evenly differ by several orders of magnitude, otherwise, the biased label values will have a negative effect on the model performance.

### 3.2 Rational analysis of equilibrium geometry‐based descriptors

**FIGURE 4:**
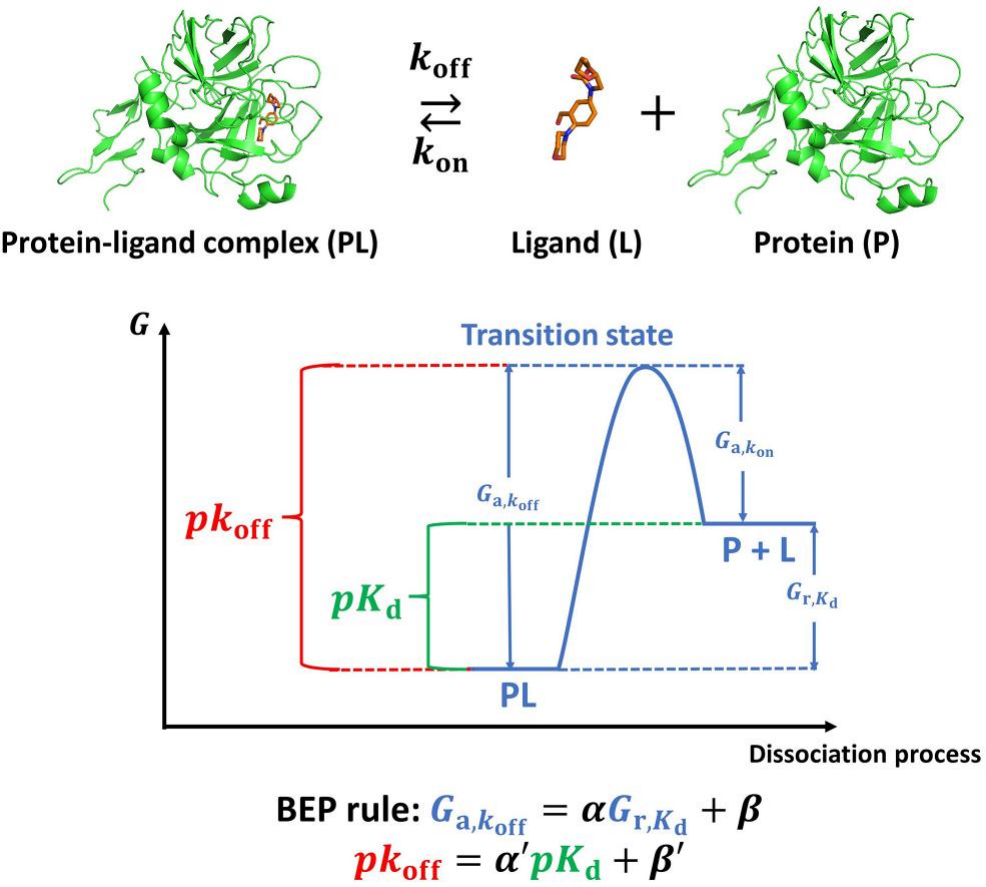
Application of the BEP relation to the binding/dissociation process of proteins and ligands.

The input representation of the developed ML models in this work is derived from the equilibrium geometry of the protein–ligand complex, while the prediction of *pk*_off_ needs the information in the transition state along the dissociate kinetic pathway. It is necessary to investigate the possibility of *pk*_off_ prediction by characterizing the structural features of protein–ligand complexes, such as binding site interaction counts without deriving features that represent the dissociation process itself. For this, the Brønsted–Evans–Polanyi (BEP) relation (Equations (8) and (9)) is applied to the binding/dissociation process of proteins and ligands (Figure 4) in this work to analyze whether it is suitable to adopt the equilibrium geometry‐based descriptors to predict *pk*_off_ for each dataset. The BEP relation was initially proposed by Brønsted, Bell, Evans and Polanyi.[^62^, ^63^, ^64^] It generally relates the activation energy (*G*_a_, a kinetic physical quantity) for a given reaction or elementary step to the corresponding reaction energy (*G*_r_, a thermodynamic physical quantity) in a linear manner via two adjustable parameters of *α* and *β*. Through using the BEP relation, it is possible to offer a more computationally efficient approach to evaluate the complex kinetic behavior of a chemical reaction based on the readily available thermodynamic data or equilibrium molecular/cluster geometries compared with the rigorous methods (e.g., the transition state theory).^[65]^ More details of the BEP relation can be found in Bronsted et al.^[66]^

(8) $$G_{a}=\alpha G_{r}+\beta$$

(9) $$pk_{\text{off}}=\alpha^{\prime }pK_{d}+\beta^{\prime }\;\left(pk_{\text{off}}=-\log\;k_{\text{off}}\propto G_{a},pK_{d}=-\log\;K_{d}\propto G_{r}\right)$$

**FIGURE 5:**
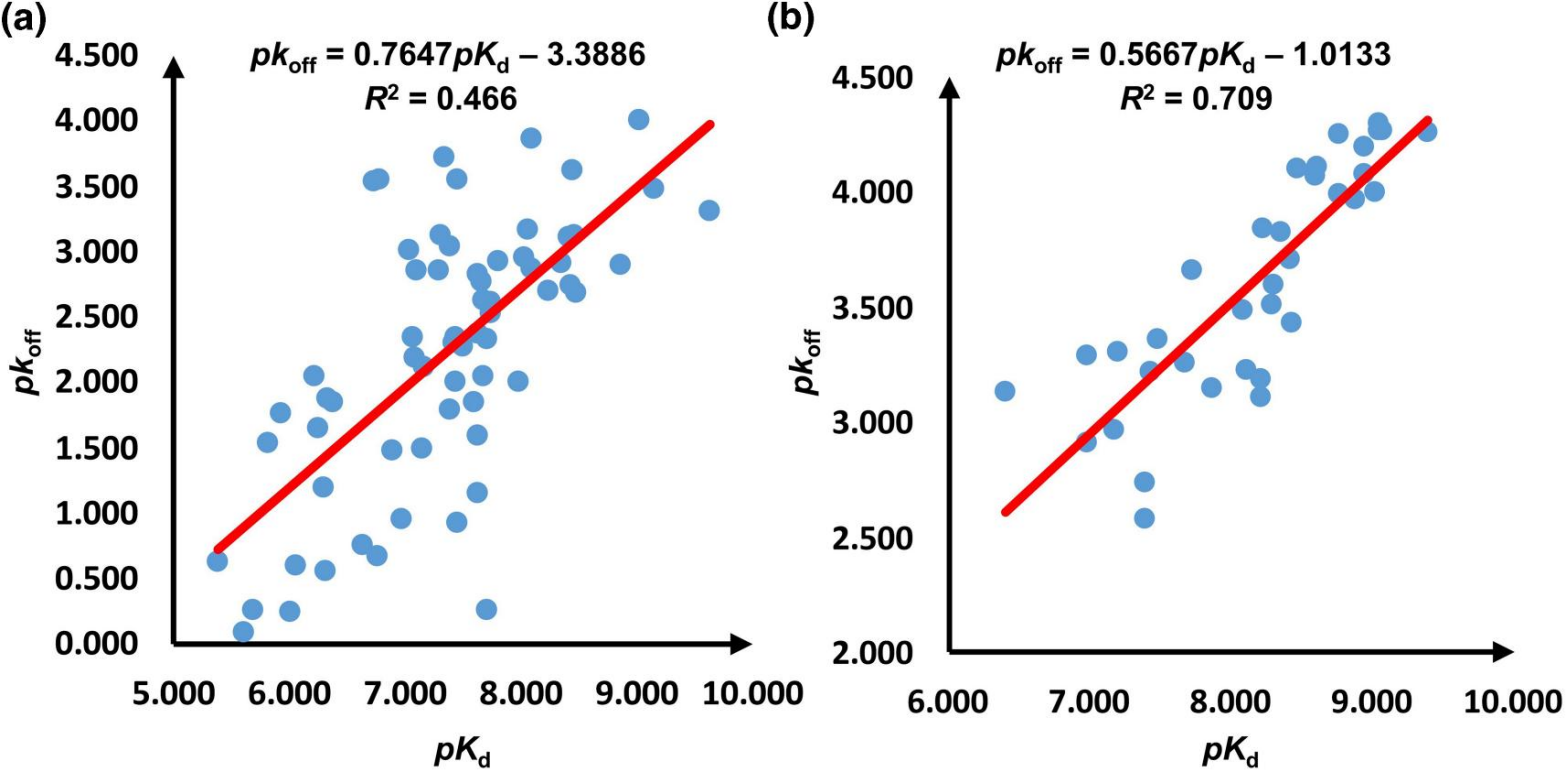
The BEP relations for the HSP90 (a) and RIP1 kinase (b) datasets.

The parameters of Equation (9) are fitted with the *pk*_off_ and *pK*_d_ values from each dataset through the least square method. The fitting results of the HSP90 and RIP1 kinase datasets are presented in Figure 5a and Figure 5b, respectively. It is seen that the *R* ^2^ of the BEP relation for the HSP90 and RIP1 kinase datasets is relatively acceptable, that is, they are linearly correlated with the binding affinity data to some extent, which indicates that it is possible to use the descriptors derived from the equilibrium geometry of the protein–ligand complex to approximately replace those derived from the transition state along the dissociate kinetic pathway to model the binding kinetics. Thus, the equilibrium geometry‐based descriptors used in this work are suitable to predict *pk*_off_ for the HSP90 and RIP1 kinase datasets.

### 3.3 Regression result comparisons of different ML models

**FIGURE 6:**
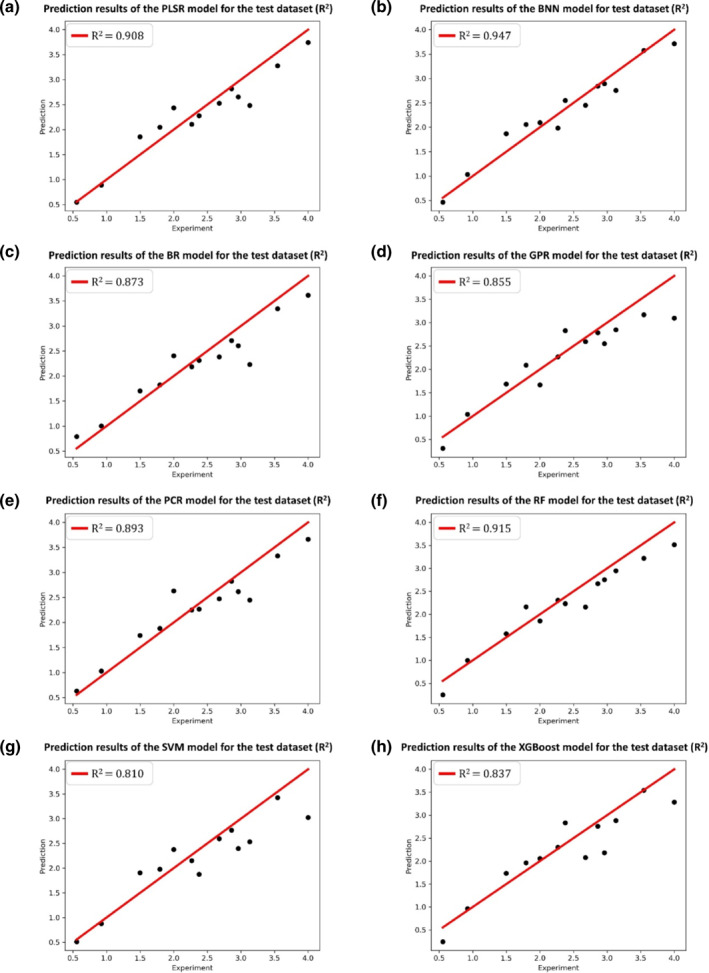
The prediction results of eight ML models for the HSP90 test set.

**FIGURE 7:**
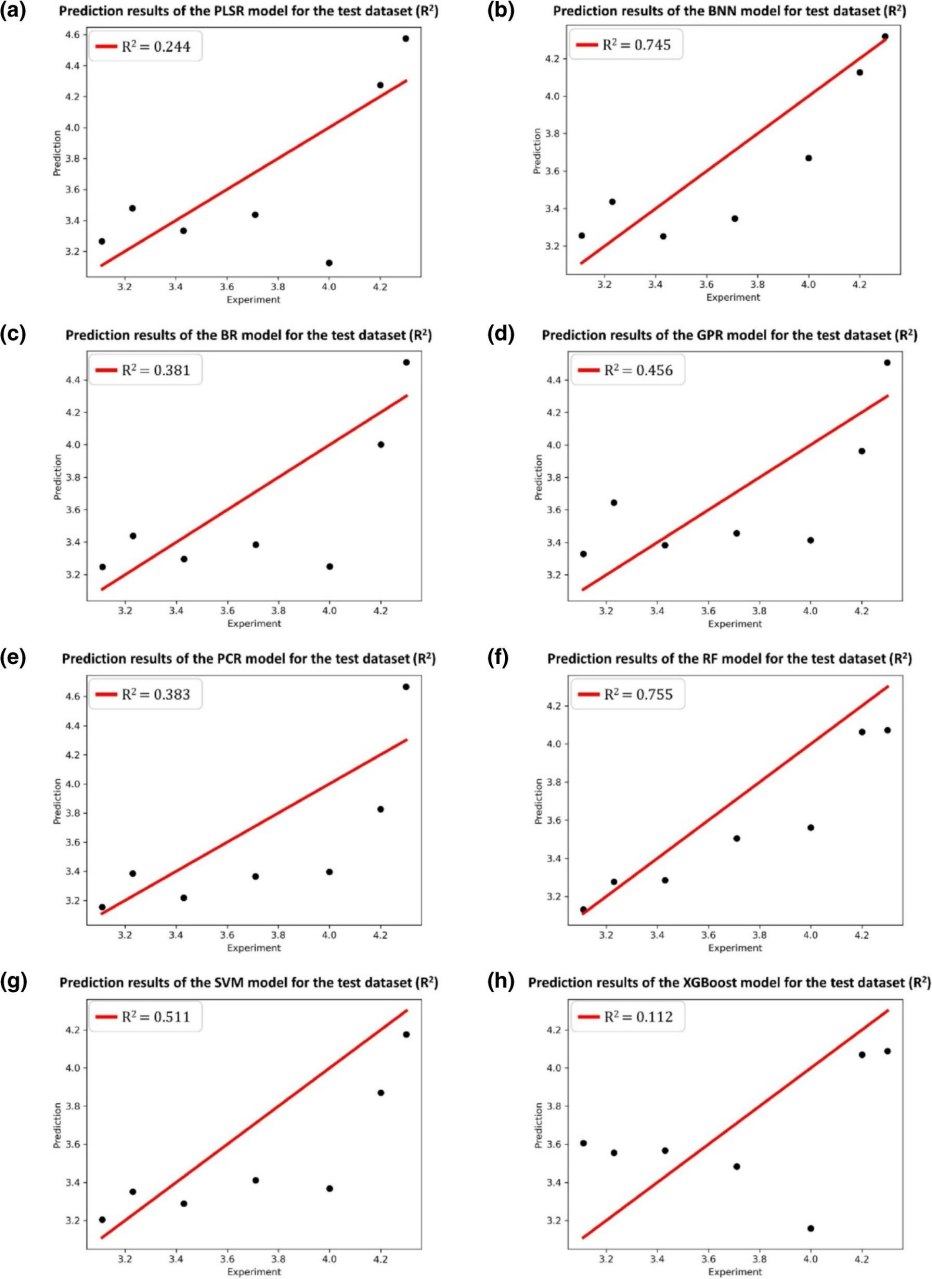
Prediction results of eight ML models for the RIP1 kinase test set.

**FIGURE 8:**
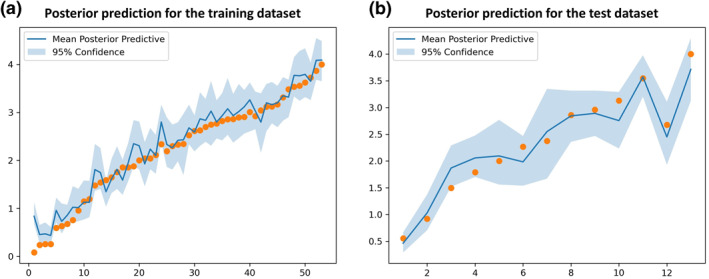
The posterior predictions of the BNN model on the (a) training and (b) test sets for the HSP90 dataset.

**FIGURE 9:**
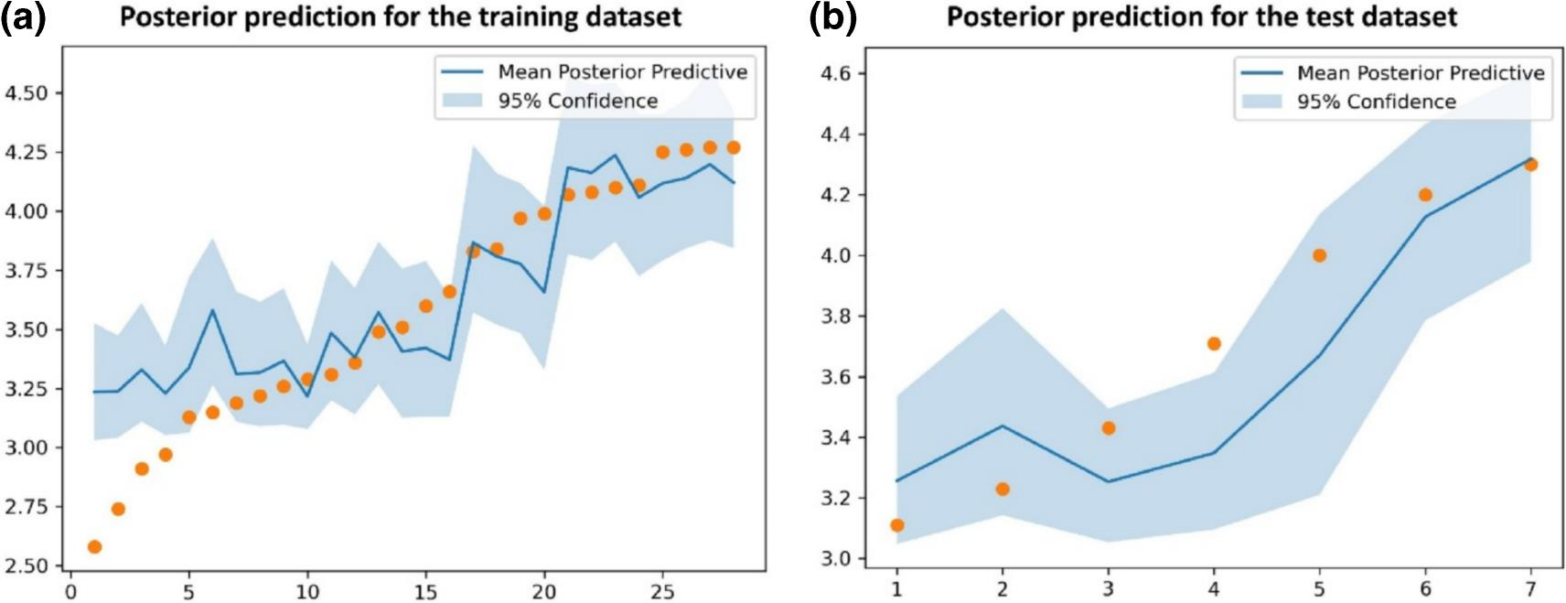
The posterior predictions of the BNN model on the (a) training and (b) test sets for the RIP1 kinase dataset.

The division results of the training and test sets for the HSP90 dataset follow the original literature^[39]^ to ensure the consistency of comparison. 66 protein–ligand complexes are divided into 53 training samples and 13 test samples with a radio of 4:1. As for the RIP1 kinase dataset, 35 protein–ligand complexes are divided into 28 training samples and seven test samples with the same radio of 4:1. In this work, the selections of optimizer, loss function, and the activation function of the BNN model are Adam, negative evidence lower bound, and Sigmoid, respectively. The selection results of other hyper‐parameters of the BNN model for the HSP90 and RIP1 kinase datasets, as well as the hyper‐parameters of other seven ML models for the two datasets can be found in Appendixes A and B of the supplementary material, respectively. The regression results of eight ML models for the HSP90 and RIP1 kinase datasets are presented in Tables 1 and 2, respectively. The prediction results of eight ML models for the HSP90 and RIP1 kinase test sets are shown in Figures 6 and 7, respectively. Figures 8 and 9 present the posterior predictions of the BNN model on the HSP90 and RIP1 kinase training/test sets, showing their interval prediction results of *pk*_off_ with 95% confidence.

**TABLE 1:** The regression results of eight ML models for the HSP90 dataset.

Criterion	PLSR	BNN	BR	GPR	PCR	RF	SVM	XGBoost
*R* ^2^_train_	0.858	0.955	0.796	0.972	0.815	0.923	0.865	0.999
*MAE*_train_	0.302	0.172	0.380	0.123	0.353	0.203	0.270	0.017
*r*_train_	0.926	0.985	0.893	0.987	0.903	0.962	0.933	1.000
*RMSE*_train_	0.388	0.218	0.465	0.172	0.443	0.285	0.378	0.025
*Q* ^2^_cv_	0.753	0.647	0.712	0.563	0.733	0.582	0.632	0.531
*MAE*_cv_	0.402	0.458	0.452	0.528	0.431	0.511	0.509	0.544
*r*_cv_	0.869	0.808	0.844	0.756	0.856	0.764	0.795	0.734
*RMSE*_cv_	0.511	0.612	0.553	0.680	0.532	0.665	0.624	0.705
*R* ^2^_test_	0.908	0.947	0.873	0.855	0.893	0.915	0.810	0.837
*MAE*_test_	0.232	0.184	0.261	0.289	0.238	0.236	0.316	0.288
*r*_test_	0.961	0.976	0.955	0.938	0.954	0.972	0.925	0.926
*RMSE*_test_	0.290	0.220	0.341	0.365	0.314	0.279	0.418	0.387

**TABLE 2:** Regression results of eight ML models for the RIP1 kinase dataset.

Criterion	PLSR	BNN	BR	GPR	PCR	RF	SVM	XGBoost
*R* ^2^_train_	0.642	0.767	0.711	0.977	0.770	0.880	0.907	1.000
*MAE*_train_	0.247	0.185	0.217	0.055	0.196	0.129	0.129	0.001
*r*_train_	0.801	0.901	0.857	0.989	0.877	0.961	0.959	1.000
*RMSE*_train_	0.297	0.239	0.267	0.075	0.238	0.171	0.151	0.002
*Q* ^2^_cv_	0.234	0.531	0.286	0.507	0.357	0.266	0.509	0.382
*MAE*_cv_	0.369	0.274	0.347	0.255	0.308	0.342	0.284	0.314
*r*_cv_	0.549	0.742	0.543	0.731	0.624	0.521	0.715	0.620
*RMSE*_cv_	0.434	0.339	0.419	0.348	0.397	0.425	0.347	0.389
*R* ^2^_test_	0.244	0.745	0.381	0.456	0.383	0.755	0.511	0.112
*MAE*_test_	0.285	0.188	0.280	0.281	0.300	0.174	0.249	0.338
*r*_test_	0.701	0.884	0.735	0.709	0.774	0.950	0.836	0.445
*RMSE*_test_	0.381	0.221	0.344	0.323	0.344	0.217	0.306	0.412

From Tables 1, 2 and Figures 6 and 7, it is seen that the overall performances of the BNN models for both HSP90 and RIP1 kinase proteins outperform other seven ML models in terms of the evaluation criteria of *R* ^2^, $Q_{\text{cv}}^{2}$, *MAE*, *r*, and *RMSE*. Specifically, for the HSP90 protein, Table 1 shows that the results of the leave‐one‐out cross‐validation method ($Q_{\text{cv}}^{2}$) for all models exceed 0.5, indicating that these models have certain extrapolation abilities for the HSP90 dataset. However, the nonlinear GPR and XGBoost models exist the overfitting issue to some extent according to their $R_{\text{train}}^{2}$ and $R_{\text{test}}^{2}$, highlighting the advantage of the BNN model in avoiding the overfitting issue owe to its interval prediction ability. For the RIP1 kinase protein, Table 2 shows that the results of the leave‐one‐out cross‐validation method ($Q_{\text{cv}}^{2}$) for the BNN model exceed 0.5 and there is no overfitting issue in the BNN model. Nevertheless, all other ML models have more or less overfitting issues according to their $R_{\text{train}}^{2}$ and $R_{\text{test}}^{2}$, demonstrating the superiority of the BNN model in small data sample modeling as the sample number of the RIP1 kinase dataset is fewer than that of the HSP90 dataset. From Table 1 and Figure 6 it is found that the top ranked BNN model ($R_{\text{test}}^{2}=0.947$, *MAE*_test_ = 0.184, *r*_test_ = 0.976, *RMSE*_test_ = 0.220) has larger $R_{\text{test}}^{2}$, *r*_test_ and lower *MAE*_test_, *RMSE*_test_ compared with the commonly used PLSR model ($R_{\text{test}}^{2}=0.908$, *MAE*_test_ = 0.232, *r*_test_ = 0.961, *RMSE*_test_ = 0.290), and outperforms other ML models in terms of $R_{\text{test}}^{2}$, which indicates the powerful ability of the BNN method in modeling the binding kinetics of HSP90 inhibitors. Figure 8 shows that almost all the data points of the test set are located within the prediction range of 95% confidence, which also verifies the strong robustness of the BNN model in predicting the *pk*_off_ of HSP90 inhibitors. However, the prediction accuracy of the BNN model on the RIP1 kinase test set ($R_{\text{test}}^{2}=0.745$, *MAE*_test_ = 0.188, *r*_test_ = 0.884, *RMSE*_test_ = 0.221) (Table 2 and Figure 7) and the posterior predictions of the BNN model on the RIP1 kinase training/test sets (Figure 9) are not as great as those on the HSP90 test set, which is mainly attributed to the characteristics of the RIP1 kinase dataset with respect to its sparse distribution of the ligands in chemical space and biased distribution of *pk*_off_ values (as discussed in Subsection 3.1). Despite that, the BNN model for the RIP1 kinase dataset still outperforms other ML models in terms of *Q* ^2^_cv_, $R_{\text{test}}^{2}$, *MAE*_test_, *r*_test_, and *RMSE*_test_, as shown in Table 2 and Figure 7. In fact, the prediction accuracy of $R_{\text{test}}^{2}=0.745$ for the RIP1 kinase protein is acceptable for screening ligands as most of the binding kinetic models have the prediction accuracy ($R_{\text{test}}^{2}$) generally ranging from 0.7 to 0.8.[^15^, ^36^, ^39^] The above comparably better results of the BNN model benefit from the superiority of the BNN ability in nonlinear fitting, interval prediction and small data sample modeling (more detailed discussions are given in Subsection 3.5). More details of the prediction results of eight ML models for the HSP90 and RIP1 kinase training sets are given in Figure A1 in Appendix A and Figure B1 in Appendix B of the supplementary material, respectively.

### 3.4 Performance comparisons between the BNN model and other existing models

To better highlight the superiority of the prediction accuracy of our BNN model in predicting the *pk*_off_ of HSP90 inhibitors, another binding kinetic model existed in literature is collected and compared with our BNN model in terms of *R* ^2^, *Q* ^2^_cv_, *MAE*, *r*, *RMSE*, as shown in Table 3. Note that there is no model comparison for the RIP1 kinase dataset as it has not been studied by other existing models to the best of our knowledge.

**TABLE 3:** Performance comparisons between the BNN model and the existing model in the literature for the HSP90 dataset.

Criterion	BNN (this work)	gCOMBINE^b^ ^[39]^
Training/test sample number	53/13	53/13
*R* ^2^_train_	0.955	0.858
*MAE*_train_	0.172	0.302
*r*_train_	0.985	0.926
*RMSE*_train_	0.218	0.388
*Q* ^2^_cv_	0.647^a^	0.753^**a**^
*MAE*_cv_	0.458	0.402
*r*_cv_	0.808	0.869
*RMSE*_cv_	0.612	0.511
*R* ^2^_test_	0.947	0.908
*MAE*_test_	0.184	0.232
*r*_test_	0.976	0.961
*RMSE*_test_	0.220	0.290

^a^ Leave‐one‐out cross‐validation.

^b^ Three problematic *pk*_off_ values in literature^[39]^ are corrected according to the literature.^[31]^

Ganotra et al.^[39]^ used the descriptors of van der Waals and electrostatic interaction energies calculated by the gCOMBINE software^[43]^ to obtain the PLSR model for predictions of *pk*_off_ based on a training set of 53 inhibitors and a test set of 13 inhibitors. Three problematic *pk*_off_ values in Ganotra's work are corrected according to the previous literature.^[31]^ Table 3 shows that our BNN model outperforms this binding kinetic model developed in the literature in terms of *R* ^2^_test_, *MAE*_test_, *r*_test_, and *RMSE*_test_ to some extent for the HSP90 dataset.

### 3.5 The analyses of the BNN model achieving the best performance

Compared with the mechanism modeling method, the ML modeling methods (e.g., BNN, etc.) are able to reduce the noises caused by the inaccuracies in the potential energy functions and molecular models, and can identify the mechanistically important interaction terms.^[37]^

Compared with other ML models, better performance of the BNN model is mainly attributed to the following reasons:

(1) The relationship between the van der Waals/electrostatic interaction energies and *pk*_off_ is relatively complex and not simply linear, which is a possible reason why the linear ML models developed in this work perform worse than the BNN model. BNN is expert at fitting nonlinear relationships between descriptors and labels, making it possible to process the complex correlations between van der Waals/electrostatic interaction energies and *pk*_off_.
(2) The BNN model outperforms other nonlinear models developed in this work because it is a probabilistic method that produces a full distribution of the function output as opposed to the ordinary nonlinear model that provides point estimates; that is, it is able to provide interval predictions and developments of trustworthy models. In addition, BNN statistics offer a natural way to reason for uncertainty in predictions.^[67]^ The uncertainty is more consistent with the observed errors,^[68]^ leading to the results that the BNN model is more robust and less often overconfident or underconfident compared with other non‐Bayesian counterparts.^[69]^
(3) In the case study of the RIP1 kinase dataset that has fewer data samples than the HSP90 dataset, only the BNN model has acceptable regression results, while all other ML models developed in this work have nearly overfitting issues. The obtained results may be attributed to the reason that BNN is very data‐efficient and capable of learning from a small dataset without overfitting.^[70]^ This characteristic mainly owes to its ability in distinguishing between epistemic uncertainty (i.e., uncertainty caused by a lack of understanding of the optimal model) and aleatoric uncertainty (i.e., uncertainty due to the random nature and the unpredictable of the studied physical system),^[71]^ as well as its capability for considering the uncertainty in predictions.

## 4 CONCLUSION

It is well known that quantitative predictions of dissociation rate constants (*k*_off_) have always been a challenging problem in the field of computational chemistry. This work aims to investigate the possibility of predictions of *k*_off_ for protein–ligand complexes using a novel binding kinetic modeling methodology based on eight ML methods and the descriptors of the van der Waals/electrostatic interaction energies. Two case studies involving the HSP90 and RIP1 kinase proteins are used to test the proposed methodology. The outstanding regression result of the BNN model for the HSP90 dataset indicates that the BNN model is more robust and accurate in predicting the *pk*_off_ of HSP90 inhibitors compared with the commonly used PLSR model (BNN: $R_{\text{test}}^{2}=0.947$, *MAE*_test_ = 0.184, *r*_test_ = 0.976, *RMSE*_test_ = 0.220; PLSR: $R_{\text{test}}^{2}=0.908$, *MAE*_test_ = 0.232, *r*_test_ = 0.961, *RMSE*_test_ = 0.290), and also outperforms other six typical ML models (BR, GPR, PCR, RF, SVM, XGBoost). Although the regression results of the RIP1 kinase dataset are not as good as those of the HSP90 dataset, the BNN model ($R_{\text{test}}^{2}=0.745$, *MAE*_test_ = 0.188, *r*_test_ = 0.884, *RMSE*_test_ = 0.221) still outperforms other ML models in terms of *Q* ^2^_cv_, $R_{\text{test}}^{2}$, *MAE*_test_, *r*_test_, and *RMSE*_test_ for the RIP1 kinase dataset. Overall, this study demonstrates that the developed BNN model has a great ability in estimating *pk*_off_ due to its superiority in nonlinear fitting, interval prediction and small data sample modeling. Future work will consider the computationally efficient AM1‐BCC charges and further integrate our BNN‐based binding kinetic model with high‐throughput drug discovery tasks such as drug screenings in large‐scale databases or optimization‐based *de novo* drug designs.

## CONFLICT OF INTEREST STATEMENT

There is no conflict of interest to declare.

## ETHICS STATEMENT

There is no potential conflict of interest. Research has not involved Human Participants and/or Animals. The material, which is presented in this paper has not been published or submitted elsewhere. All authors confirm that they have read and approved the paper, that they have met the criteria for authorship, that they believe that the paper represents honest work, and that they are able to verify the validity of the results reported. If accepted, it will not be published elsewhere in the same form, in either the same or another language, without the consent of the editors and the publisher. Reference should be made to previously published abstracts, etc. in the introductory section. Responsibility for the accuracy of the material in the paper, including bibliographic citations, lies entirely with the authors.

## Supporting information

Supporting Information S1

## Data Availability

The data that support the findings of this study are openly available in Ganotra et al. at https://doi.org/10.1021/acsmedchemlett.8b00397, reference number [39], and Schuetz et al. at https://doi.org/10.1039/D0MD00178C, reference number [49].
