# Supplementary material for: Machine learning methods for developments of binding kinetic models in predicting protein‐ligand dissociation rate constants
Source: Smart Mol. 2023 Nov 10;1(3):e20230012. doi: 10.1002/smo.20230012 (PMC12118191; doi:10.1002/smo.20230012)
Supplement: Supplementary file 1 — Supporting Information S1 [file SMO2-1-e20230012-s001.docx]

**Machine Learning Methods for Developments of Binding Kinetic Models in Predicting Protein-Ligand Dissociation Rate Constants**

**Yujing Zhao^a^, Qilei Liu^a*^, Jian Du^a^, Qingwei Meng^a,b^, Lei Zhang^a*^**

^a^State Key Laboratory of Fine Chemical, Frontiers Science Center for Smart Materials Oriented Chemical Engineering, Institute of Chemical Process Systems Engineering, School of Chemical Engineering, Dalian University of Technology, Dalian 116024, China

^b^Ningbo Institute of Dalian University of Technology, Ningbo 315016, China

*Corresponding authors.

E-mail addresses: Qilei Liu (liuqilei@dlut.edu.cn), Lei Zhang (keleiz@dlut.edu.cn).

**Appendix A**

Whether the standardization scaling method is used, and the hyper-parameters of eight ML algorithms for the HSP90 dataset are presented as follows:

**PLSR**: the standardization scaling method is used; the number of principal components is 4; the threshold of standard deviation is 0.40.

**BNN**: the standardization scaling method is not used; the epoch is 500; the learning rate is 0.1; the prior variance is 10; the number of principal components is 20; the number of neurons in the hidden layer is 20; the threshold of standard deviation is 0.4; the dropout rate is 0.1.

**BR**: the standardization scaling method is used; the number of principal components is 7; the threshold of standard deviation is 0.35.

**GPR**: the standardization scaling method is not used; the number of principal components is 4; the threshold of standard deviation is 0.25; the kernel combines the linear and radial basis functions with alpha = 0.1, n_restarts_optimizer = 5, and normalize_y = False.

**PCR**: the standardization scaling method is used; the number of principal components is 8; the threshold of standard deviation is 0.35.

**RF**: the standardization scaling method is not used; the number of principal components is 8; the threshold of standard deviation is 0.30; n_estimators = 8.

**SVM**: the standardization scaling method is used; the number of principal components is 9; the threshold of standard deviation is 0.05; the kernel is the radial basis function with C = 2.4.

**XGBoost**: the standardization scaling method is not used; the number of principal components is 8; the threshold of standard deviation is 0.24; max_depth = 5; learning_rate = 0.2, n_estimators =30.


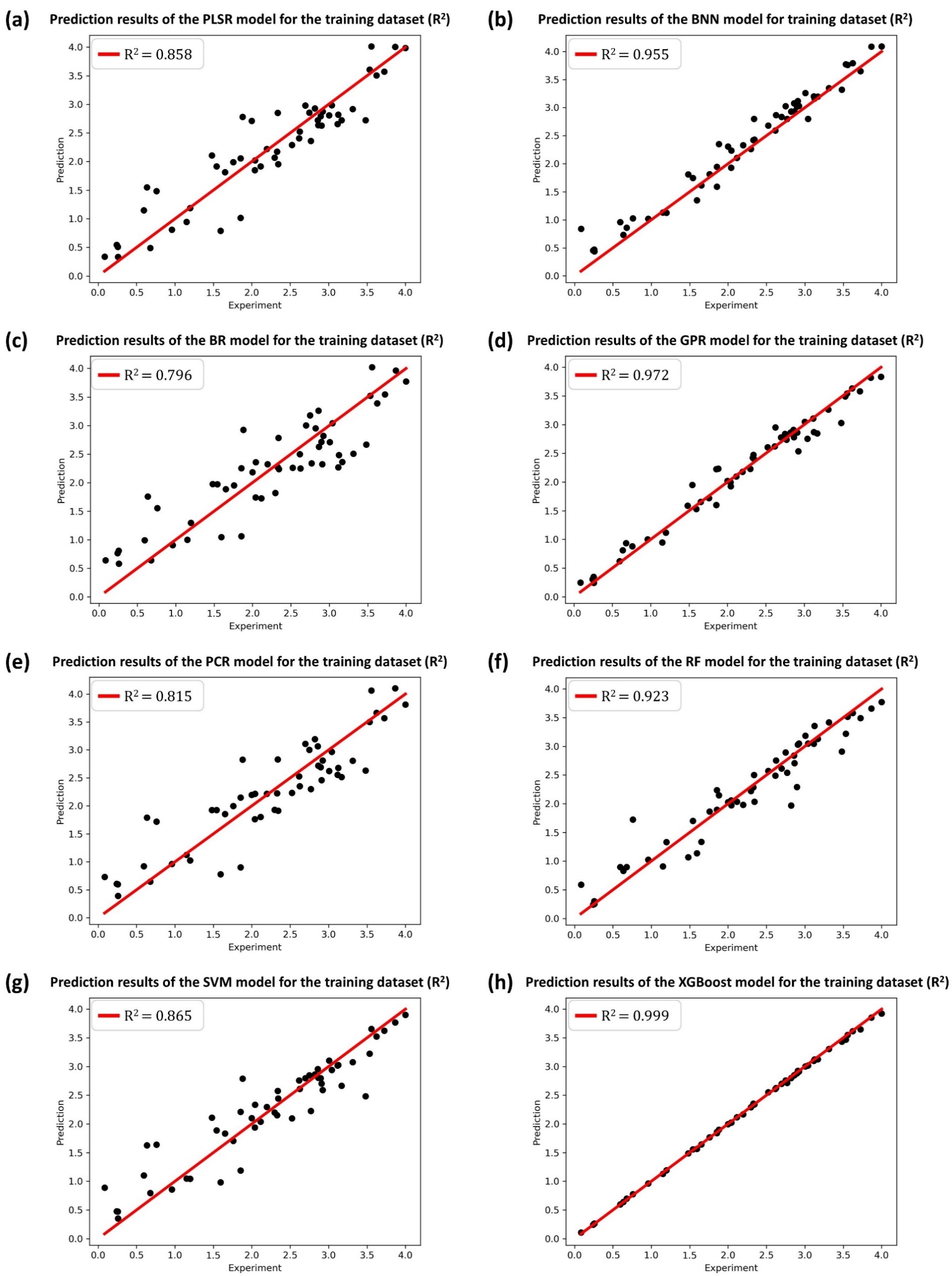


**Figure A1** The prediction results of eight ML models for the HSP90 training set.

**Appendix B**

Whether the standardization scaling method is used, and the hyper-parameters of eight ML algorithms for the RIP1 kinase dataset are presented as follows:

**PLSR**: the standardization scaling method is not used; the number of principal components is 3; the threshold of standard deviation is 0.45.

**BNN**: the standardization scaling method is not used; the epoch is 500; the learning rate is 0.1; the prior variance is 10; the number of principal components is 20; the number of neurons in the hidden layer is 17; the threshold of standard deviation is 0.05; the dropout rate is 0.3.

**BR**: the standardization scaling method is not used; the number of principal components is 10; the threshold of standard deviation is 0.30.

**GPR**: the standardization scaling method is not used; the number of principal components is 10; the threshold of standard deviation is 0.20; the kernel combines the linear and radial basis functions with alpha = 0.1, n_restarts_optimizer = 5, and normalize_y = True.

**PCR**: the standardization scaling method is not used; the number of principal components is 10; the threshold of standard deviation is 0.30.

**RF**: the standardization scaling method is used; the number of principal components is 11; the threshold of standard deviation is 0.40; n_estimators = 8.

**SVM**: the standardization scaling method is not used; the number of principal components is 11; the threshold of standard deviation is 0.35; the kernel is the radial basis function with C = 2.7.

**XGBoost**: the standardization scaling method is not used; the number of principal components is 10; the threshold of standard deviation is 0.35; max_depth = 6; learning_rate = 0.3, n_estimators =30.


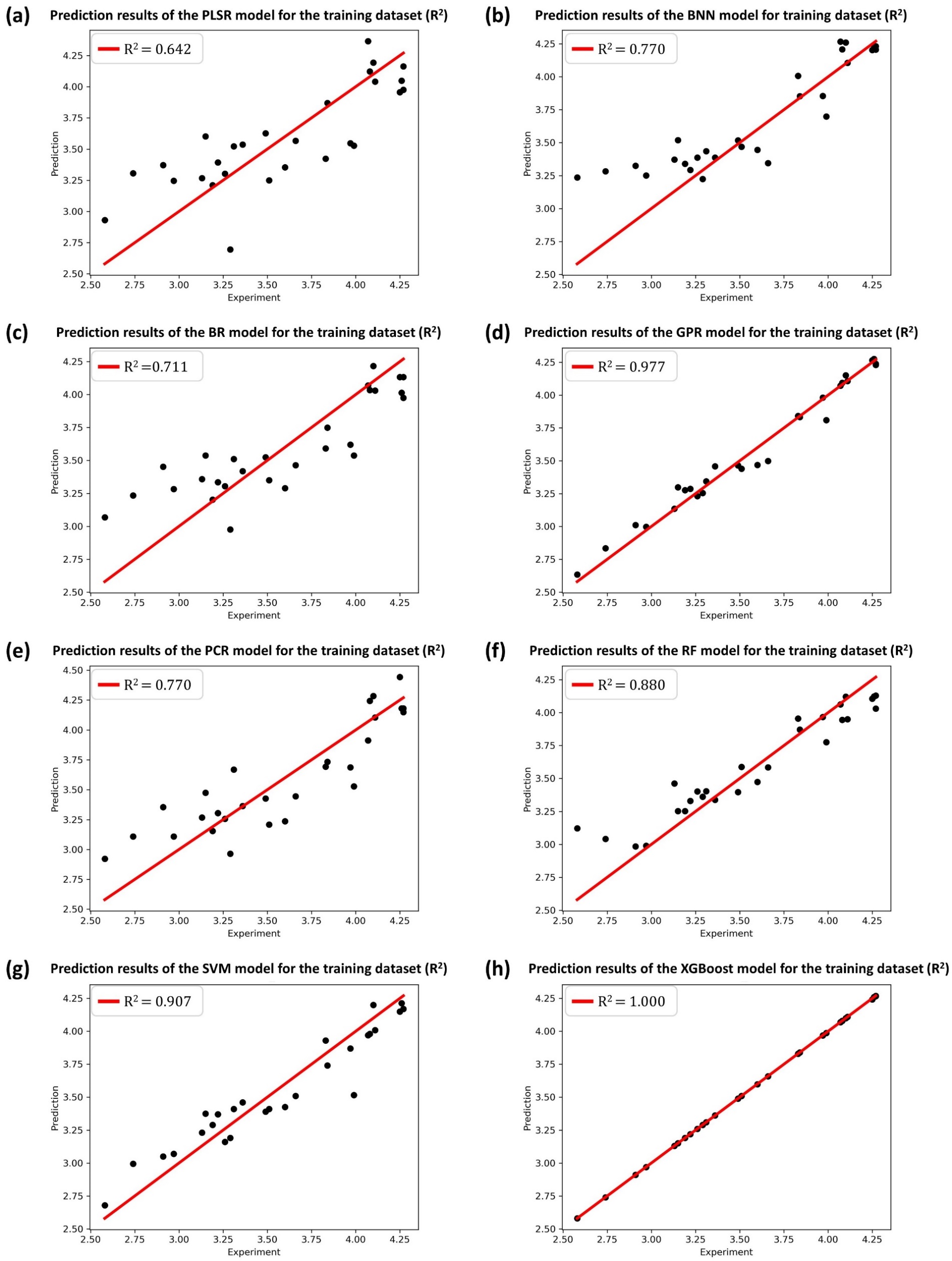


**Figure** **B1** The prediction results of eight ML models for the RIP1 kinase training set.
